# EXPLORATION OF PREPAREDNESS FOR MEDICAL DECISION MAKING FOR FAMILY CAREGIVERS OF PEOPLE WITH DEMENTIA

**DOI:** 10.1093/geroni/igad104.0042

**Published:** 2023-12-21

**Authors:** Peiyuan Zhang, John Cagle

**Affiliations:** University of Maryland, Baltimore, Baltimore, Maryland, United States; University of Maryland, Baltimore, Baltimore, Maryland, United States

## Abstract

As dementia progresses, many family caregivers of people with dementia (PWD) often experience increasing demands of making medical decisions for their loved ones and feel unprepared, causing severe psychological distress. This presentation explores what factors influence preparedness level of family caregivers in medical decision-making for PWD. As part of a larger two-group randomized trial in which 273 PWD-family caregiver dyads received either a control protocol or a multi-component communication intervention including advance care planning (ACP) facilitation, this qualitative study used thematic analysis methods to analyze 10 transcripts resulting from audio-recorded and transcribed ACP conversations with an ACP facilitator, PWD, and family caregivers. Interview duration was over 30 minutes. The major themes identified were: (1) knowledge about PWD’s treatment preferences, (2) experience with death and dying, (3) previous decision-making involvement, and (4) spiritual beliefs. Many family caregivers found ACP conversations with PWD about their goals of care to be beneficial in improving their confidence in making medical decisions. A few participants also reported witnessing a loved one’s suffering at the end of life or having made care decisions for family before helped them to initiate conversations with PWD about medical wishes, thus enhanced readiness for decision making. Lastly, few caregivers reported belief in God would help them make the “right decisions”. Preliminary results highlight that caregivers’ understanding of PWD’s treatment preferences helps to prepare family caregivers for decision-making, underlining the need for early and culturally appropriate ACP conversations among residents, caregivers, and clinicians.

